# Prevalence of chronic obstructive pulmonary disease (COPD) among rheumatoid arthritis: results from national inpatient database

**DOI:** 10.1080/20009666.2018.1485460

**Published:** 2018-08-23

**Authors:** Rashmi Dhital, Sijan Basnet, Prakash Paudel, Yam Prasad Acharya, Dilli Ram Poudel

**Affiliations:** a Internal Medicine, Tower Health System, West Reading, PA, USA; b Internal Medicine, Berkshire Medical Center, Pittsfield, MA, USA; c Internal Medicine, Miriam Hospital, Providence, RI, USA

**Keywords:** Rheumatoid arthritis, RA, chronic obstructive pulmonary disease, COPD, association, national database, National Inpatient Sample, NIS, HCUP

## Abstract

Rheumatoid arthritis (RA) is being increasingly recognized as an important contributor to chronic obstructive pulmonary disease (COPD). Although smoking is a major risk factor, other factors may play a role. We used National Inpatient Sample (NIS) from 2013 to explore this relationship. We used propensity matching with a 1:3 nearest-neighbor-matching algorithm to match 1 RA hospitalization to 3 age- and-sex-matched comparators. In the age- and-sex-matched population, RA had a higher odds of COPD (OR 1.20, 95% CI: 1.17–1.22, *p* < 0.0001).

RA is associated with increased COPD prevalence, independent of smoking. COPD might fall within the spectrum of RA complications, likely due to autoimmune and inflammatory mechanisms.

## 1. Introduction

Chronic obstructive pulmonary disease (COPD) refers to the limitation in airflow that is not fully reversible [1]. There is a growing body of literature suggesting that rheumatoid arthritis (RA) is associated with an increased risk of incident COPD compared to matched general population. Studies have been carried out in various regions of the world- Canada, Israel, Taiwan, Netherlands, Sweden and Minnesota, all suggesting that COPD is more prevalent in RA [2–7]. Although smoking is known to be a major risk factor for development and progression of COPD, the presence of substantial number of COPD patients who have never smoked suggests that other factors may play a role, with inflammatory and immune responses being important ones [8,9]. We aimed to explore this relationship in an inpatient setting, using a large inpatient database.

## 2. Material and methods

National Inpatient Sample (NIS) is the largest all- payer publicly available inpatient care database in the US, which is a part of Healthcare cost and Utilization Project (HCUP), and is sponsored by Agency of Healthcare Research and Quality (AHRQ). It is a stratified 20% sample of all discharges from HCUP participating hospitals and contains information on over 7 million hospital discharges per year [10]. We selected all adults (≥18 years) discharged from 1 January 2013 to 31 December 2013. We developed two age- and sex-matched groups of patients: one with a diagnosis of RA based on International Classification of Disease (ICD-9) code(s) 714.x (714.0, 714.1, 714.2, 714.30, 714.31, 714.32, 714.33 and 714.81) and the other without a diagnosis of RA. Matching was done with a 1:3 nearest-neighbor-matching algorithm. Patients who had a diagnosis of COPD were identified based on ICD-9 codes 491.x, 492.x and 496 (491.2, 491.20, 491.21, 491.22, 492, 4920, 4928 and 496). We calculated the odds of association of RA with COPD by univariate and multivariate (controlling for covariates such as age, sex, race and smoking status) analyses. Smoking status was identified based on ICD-9 codes for current smoking as well as a history of smoking. We used STATA version 13.0 for analysis.

Ethical clearance and patient consent was not sought as the NIS HCUP database contains de-identified patient data.

## 3. Results

Baseline demographic characteristics of the hospitalization groups before and after matching are listed in Table 1. In the original (unmatched) group of 29,922,246 hospitalizations, RA (468,750; 1.57%) was more common in Caucasians (76%) and females (73.98%) with a mean age of 67.47 years. The rate of smoking was higher in RA hospitalizations compared to those without RA (28.88% vs 26.09%, *p* < 0.0001) and the COPD prevalence was higher (21.12% vs 13.41%, *p* < 0.0001). RA was associated with an increased COPD prevalence, with an odds ratio (OR) of 1.28 (95% CI 1.26–1.31, *p* < 0.0001), even after multivariate adjustment (Table 2).

**Table 1.** Demographic characteristics of rheumatoid arthritis hospitalizations compared to those without rheumatoid arthritis from the National Inpatient Sample (2013).

		Before nearest-neighbor propensity matching				After nearest-neighbor propensity matching		
	Total	RA hospitalizations	Non-RA hospitalizations	*p*-Value	Total	RA hospitalizations	Non-RA hospitalizations	*p*-Value
No of observations in the sample (*n*)	5,984,453	93,750	5,890,703		375,000	93,750	281,250	
Weighted estimate, *N* (%)	29,922,246 (98.43)	468,750 (1.57)	29,453,496 (98.43)		1,874,999	468,750 (25)	1,406,249 (75)	
Sex, *N* (%)								1
Male	12,270,187 (41.01)	121,965 (26.02)	12,148,222 (41.25)	<0.0001	487,860 (26.02)	121,965 (26.02)	365,895 (26.02)	
Female	17,652,059 (58.99)	346,785 (73.98)	17,305,274 (58.75)		1,387,139 (73.98)	346,785 (73.98)	1,040,354 (73.98)	
Age								
Mean (se)		67.46 (0.08)	57.19 (0.10)	<0.0001		67.47 (0.06)	67.47 (0.08)	1
Age Category, %				<0.0001				
18–34	20.07	3.00	20.34		3.00	3.00	3.00	1
35–64	39.29	35.82	39.35		35.82	35.82	35.81	
>65	40.64	61.19	40.31		61.19	61.19	61.19	
Race, %				<0.0001				<0.0001
Caucasians	68.67	76	68.55		73.83	76	73.11	
African American	14.73	11.95	14.78		13.04	11.95	13.40	
Others	16.6	12.05	16.67		13.13	12.05	13.49	
Charlson Comorbidity Index, %				<0.0001				<0.0001
0	41.35	0.76	42		22.58	0.76	29.85	
1	20.67	29.78	20.53		24.99	29.78	23.39	
≥2	37.98	69.46	37.47		52.43	69.46	46.76	
Smoking hx %	26.13	28.88	26.09	<0.0001	26.94	28.88	26.30	<0.0001
COPD prevalence, %	13.53	21.12	13.41	<0.0001	18.72	21.12	17.92	<0.0001

**Table 2.** Univariate and multivariate logistic regression analyses for prevalence of chronic obstructive pulmonary disease (COPD) in patients with rheumatoid arthritis (RA).

	Unadjusted OR	Adjusted* OR
Unmatched population (before near-neighbor propensity matching)	1.73 (95% CI 1.70–1.76), *p* < 0.0001	1.28 (95% CI 1.26–1.31), *p* < 0.0001
Matched population (after near-neighbor propensity matching)	1.23 (95% CI 1.20–1.25), *p* < 0.0001	1.20 (95% CI 1.17–1.22), *p* < 0.0001

* Adjusted for age, gender, race and smoking status. CI: confidence interval; OR: odds ratio.

In the age and sex-matched population, we estimated a total of 1,874,999 hospitalizations in 2013; 468,750 (25%) had RA and 1,406,249 (75%) made up the comparator non-RA group. On univariate analysis, RA hospitalizations had a 1.23 (95% CI 1.20–1.25, *p* < 0.0001) times higher odds of having COPD. Multivariate logistic model demonstrated that RA was still associated with COPD, after controlling for covariates including age, sex, race and smoking status, with an OR of 1.20 (95% CI 1.17–1.22, *p* < 0.0001) (Table 2). Smoking, by itself, was associated with a significantly higher odds of COPD (aOR 3.82, 95% CI 3.74–3.91) in the adjusted analysis.

## 4. Discussion

Our findings suggest that RA is associated with an increased COPD among in-patients, independent of smoking. The mechanism of COPD in RA has been suggested by prior studies to be autoimmune or inflammatory, with cytokines and chemokines contributing to lung tissue destruction [1,8,9]. Studies looking at association of reduced lung function tests with systemic inflammatory markers have shown that COPD patients have higher levels of circulating inflammatory markers including leukocytes, fibrinogen, ESR, CRP, tumor necrosis factor-α, suggesting that systemic inflammation may have a role in COPD [11]. Also, increased production of autoantibodies against a broad spectrum of self-antigens has been demonstrated in COPD, suggesting the role of autoimmunity [12]. Authors from prior studies have also pointed out the possibility of recurrent respiratory infections in RA patients to predispose to airway obstruction [13,14].

In the setting of possible autoimmune contribution to COPD, authors have suggested that RA-related immune dysfunction could trigger abnormal inflammatory response in the airway, with higher likelihood of COPD. The other mechanism that has been postulated is that the chronic inflammation in RA may lead to alveolar wall destruction by effect on alveolar epithelial cells, ultimately leading to COPD [15] There are studies that have looked at the pulmonary function tests in patients with RA have suggested that RA patients had significantly lower values of FEV1, FVC, and FEV1/FVC- which are the indicators of obstructive lung disease [13,14]. Although restrictive lung disease (interstitial lung disease) is well recognized in RA, the association with COPD has only been evaluated by a handful of studies. Diagnosis of COPD in RA patients may often be delayed due to subtle and non-specific early symptoms, which may be further masked due to restricted mobility from joint disease. The finding of our study is in line with the conclusion by some prior studies [2–7].

The major strength of this study is the large sample size, which is representative of almost 95% of US hospitalizations. The limitations include inaccuracies in the coding inherent to the administrative databases, lack of patient level data such as quantification of smoking and the results of the pulmonary function test (PFT) tests.

## 5. Conclusion

COPD might fall within the spectrum of RA complications and one should be vigilant about diagnosing COPD early in this subpopulation. Further studies have a potential to detect if control of COPD can be established as one of the treatment targets among RA patients with COPD and help direct how early effective therapy towards joint disease may modify the subsequent risk of pulmonary complications.
